# Nitric Oxide Participates in Cold-Inhibited *Camellia sinensis* Pollen Germination and Tube Growth Partly via cGMP *In Vitro*

**DOI:** 10.1371/journal.pone.0052436

**Published:** 2012-12-18

**Authors:** Yu-Hua Wang, Xiao-Cheng Li, Qiang Zhu-Ge, Xin Jiang, Wei-Dong Wang, Wan-Ping Fang, Xuan Chen, Xing-Hui Li

**Affiliations:** 1 Tea Science Research Institute, Nanjing Agricultural University, Nanjing, China; 2 Forest Resources and Environment Institute, Nanjing Forestry University, Nanjing, China; National Taiwan University, Taiwan

## Abstract

Nitric oxide (NO) plays essential roles in many biotic and abiotic stresses in plant development procedures, including pollen tube growth. Here, effects of NO on cold stress inhibited pollen germination and tube growth in *Camellia sinensis* were investigated *in vitro*. The NO production, NO synthase (NOS)-like activity, cGMP content and proline (Pro) accumulation upon treatment with NO scavenger cPTIO, NOS inhibitor L-NNA, NO donor DEA NONOate, guanylate cyclase (GC) inhibitor ODQ or phosphodiesterase (PDE) inhibitor Viagra at 25°C (control) or 4°C were analyzed. Exposure to 4°C for 2 h reduced pollen germination and tube growth along with increase of NOS-like activity, NO production and cGMP content in pollen tubes. DEA NONOate treatment inhibited pollen germination and tube growth in a dose-dependent manner under control and reinforced the inhibition under cold stress, during which NO production and cGMP content promoted in pollen tubes. L-NNA and cPTIO markedly reduced the generation of NO induced by cold or NO donor along with partly reverse of cold- or NO donor-inhibited pollen germination and tube growth. Furthermore, ODQ reduced the cGMP content under cold stress and NO donor treatment in pollen tubes. Meanwhile, ODQ disrupted the reinforcement of NO donor on the inhibition of pollen germination and tube growth under cold condition. Additionally, Pro accumulation of pollen tubes was reduced by ODQ compared with that receiving NO donor under cold or control condition. Effects of cPTIO and L-NNA in improving cold-treated pollen germination and pollen tube growth could be lowered by Viagra. Moreover, the inhibitory effects of cPTIO and L-NNA on Pro accumulation were partly reversed by Viagra. These data suggest that NO production from NOS-like enzyme reaction decreased the cold-responsive pollen germination, inhibited tube growth and reduced Pro accumulation, partly via cGMP signaling pathway in *C. sinensis*.

## Introduction

Many plants have to cope with low temperature during their lifecycle and many physiological and molecular changes occur during cold acclimation [1]. Low temperature is also a major factor that significantly constrains the geographical distribution of plants and agricultural productivity, especially, leads to low quality tea production, and ultimately affects the economic benefits of tea production [2]. Additionally, pollen tubes as tip-growing cells are essential part of sexual reproduction in higher plants [3]. According to previous reports, exposure to cold acclimation significantly reduces the pollen germination rate [4] and alters the cellular organization in rice [5]. More interestingly, Camellia pollen germinated at low temperature (even at 3°C) on an artificial agar medium, while lily pollen did not germinate at low temperature [6]. Many genes necessary for cold acclimation in vegetative tissues were either not or weekly induced in pollen under cold stress [4], indicating that the mechanism of cold tolerance is different in vegetative and reproductive tissues [7]. All those observations suggested that the understanding of the mechanism of Camellia pollen germination and tube growth resisting to cold stress warranted serious attention.

In recent years, nitric oxide (NO) has been described as a bioactive signaling molecule playing crucial roles in many key physiological progresses in plants [8], [9]. NO is also involved in the tolerance of plants to abiotic stresses, including drought [10], high salt [11], heat [12], heavy metal [13], and UV-B radiation stress [14]. Furthermore, previous data have indicated that NO is involved in plant acclimation and freezing tolerance. For example, Zhao et al. [15] reported a marked increase in endogenous NO production in *Arabidopsis* leaves which was mainly dependent on nitric reductase activity due to up-regulating *NIA1* gene expression after exposure to 4°C. Recently, it has been demonstrated that NO negatively regulates the formation of phytosphingosine phosphate and ceramide phosphate to respond to cold exposure in *Arabidopsis* [16]. Even more, it has been revealed that NO synthase (NOS) like activity-dependent NO acts as an antioxidant or as a signal activating antioxidant defense to confer an enhanced tolerance to chilling-induced oxidative damage in *Chorispora bungeana* suspension cultured cells [17]. In addition, it is well known that higher plants accumulate free Pro in response to a number of abiotic stresses such as drought, salinity, and freezing [15]. Nevertheless only a few reports indicated that NO and Pro cross talked in cold acclimation and freezing tolerance. For example, Zhao et al. [15] showed that cold-induced NO acts as a signal to evoke Pro accumulation *via* enhanced synthesis and reduced degradation by regulating related genes of Pro biosynthetic pathway in *Arabidopsis*, which may be a function of NO in freezing tolerance. Furthermore, accumulating evidence has demonstrated that NO signaling pathway functions through the second messenger guanosine 3′, 5′-cyclic monophosphate (cGMP) in many different physiological and pharmacological responses in higher plants [18], [19]. Salmi et al. [20] reported that NO-cGMP signaling pathway played a signaling role in gravity-directed cell polarity in germinating *Ceratopteris richardii* spores, as well as guided pollen tube growth in lily (*Lilium longiflorum*) [21]. However, in many cases, NO-dependent physiological and pharmacological responses are governed by a complex signaling network, for which the biochemical and molecular mechanisms have not been deciphered [22].

Previous studies have shown that NO plays crucial roles in regulating highly polarized tip growth, especially pollen tube growth. NO participates in the growth regulation and reorientation of lily and *Arabidopsis* pollen tubes [21], [23]. In addition, NO is involved in configuration and distribution of cell wall components in *Pinus bungeana* pollen tubes by altering extracellular Ca^2+^ influx and F-actin organization [24], and partly mediate extracellular nucleotide-induced suppression of pollen germination and pollen tube elongation [25]. Furthermore, previous reports have indicated that NO plays an inhibitory role in pollen germination and tube growth in *Paulownia tomentosa* in response to UV-B light [26], and NO production in pollen tubes participates in mediating actin reorganization and programmed cell death in the self-incompatibility response of *Papaver* [9]. Over all, the response of pollen tube to cold acclimation is known, but the underlying mechanism is not. Even more, there are no data currently available to support the involvement of NO in cold acclimation in pollen tubes.

Here, we investigated the regulatory roles of NO during pollen tube elongation upon cold stress in *C. sinensis*. Specifically, we analyzed NO production in pollen tubes with or without cold stress, as well as other linked features that are essential for NO signaling pathway and cold tolerance, including cGMP, NOS-like activity and Pro accumulation.

## Materials and Methods

### Ethics Statement

All necessary permits were obtained for the described field studies from the Mausoleum of Dr. Sun Yat-sen Tea Factory of Nanjing Zhongshan Landscape Construction Group Company, China (http://www.zsyl.com/html/index.asp).

### Plant material

Mature pollen was collected from *C. sinensis* (L.) O. Kuntze trees growing in the Mausoleum of Dr. Sun Yat-sen Tea Garden, Nanjing, China. For all experiments, pollen was incubated in a control solution (containing 30 mM/L MES, 5% sucrose, 0.01% H_3_BO_3_, and 0.05% Ca(NO_3_)_2_^.^ 4H_2_O, 5% PEG4000.pH 6.0) at 25°C (control) or 4°C (cold stress) in the dark. To examine the effect of exogenous NO on pollen germination and tube growth, pollen from *C. sinensis* was germinated for 2 h in the control solution containing different concentrations of NO donor DEA NONOate (0, 25, 50, 75, 100 μM) and NO specificity was also assayed by addition of 200 μM NO scavenger 2-(4-carboxyphenyl)-4, 4, 5, 5-tetramethylimidazoline-1-oxyl-3-oxide (cPTIO) or 300 μM mammalian NO synthase (NOS) inhibitor *N*_ω_-nitro-L-arginine (L-NNA). To examine the effect of cPTIO and L-NNA on pollen germination and tube growth under cold-stress, pollen was suspended for 2 h at 4°C in the control solution containing 200 μM cPTIO or 300 μM L-NNA, respectively. To examine the effect of cGMP on DEA NONOate-modulated pollen germination and tube growth, pollen grains were germinated in cold stress for 2 h in the solution containing 25 μM DEA NONOate with or without 50 μM ODQ, respectively. To examine the effect of cGMP on cPTIO- and L-NNA-regulated pollen germination and tube growth, pollen grains were germinated in cold stress for 2 h in the solution containing 200 μM cPTIO or 300 μM L-NNA with or without 80 μM Viagra, respectively. To measure the mean tube length, at least 50 pollen tubes were detected in each of three replicates. Pollen grains were not considered germinated unless the tube length was greater than diameter of the grain.

### NO assays and NO imaging

The presence of NO in pollen tubes was assayed and visualized as previously described with small modifications [24]. After pollen grains were incubated in control solution with or without pharmacological reagents at 25°C or 4°C for 2 h, the samples were suspended in 20 μM NO-specific fluorescent probe 4-amino-5-methylamino-2′, 7′-difluorofluorescein diacetate (DAF-FM DA, Sigma) for 20 min in dark and then excess fluorophore was washed out. The specimens were examined using a 488-nm argon laser under confocal laser-scanning microscope (CLSM Leica TCSSL) with the same parameter settings. Emission signals were collected at 500–550 nm. Images acquired from the confocal microscope were analyzed with Leica Image Software and processed with Adobe Photoshop software. The relative fluorescence intensities of at least fifty pollen tubes in each of three replicates were measured using Image J, and mean relative fluorescence intensities were calculated.

To confirm the effect of cold-stress on NO generation in pollen tubes, NO production in pollen grains and tubes were measured not only by NO-specific probe but also by electron paramagnetic resonance (EPR) spectroscopy using the spin trap Fe(II)(DETC)_2_ by the method of [27], [28] with slight modification. Briefly, 2 g pollen tubes were harvested and ground in liquid N_2_ and incubated in 1 mL of buffered solution (50 mM Hepes, 1 mM DTT, 1 mM MgCl_2_, pH 7.6) for 2 min. The mixture was centrifuged at 13,200 g for 2 min. The supernatant was added to 300 μL of freshly made [Fe(II)(DETC)_2_] solution (2 M Na_2_S_2_O_4_, 6.6 mM DETC (diethyldithiocarbamate), 3.3 mM FeSO_4_, 33 mg mL^−1^ BSA), incubated for 2 min at room temperature and frozen again in liquid N_2_. EPR measurements were performed on a Bruker EMX10/12 spectrometer under following conditions: room temperature; microwave power, 19.97 mW; modulation amplitude, 3 G; time constant, 327.68 ms; scan time, 83.886 s. Representative spectra from three independent measurements were shown in the results.

### Detection of NOS-like activity

Crude extracts for NOS-like activity were prepared as previously described [15] with small modifications. Briefly, after incubated for 2 h at 25°C or 4°C, the pollen tubes were harvested and ground in liquid N_2_ together with 50 mg polyvinylpolypyrrolidone to a fine powder, and then resuspended in ice-cold extraction buffer (50 mM Tris-HCl buffer pH 7.4, 320 mM sucrose, l mM EDTA, 20 μM pepstatin, 20 μM Leupeptin, l mM PMSF and l mM DTT). Homogenate was centrifuged at 10 000 g for 30 min at 4°C and the supernatant was used as crude protein preparation. Protein concentration was determined according to Bradford's method [29] and the concentration of crude protein extracts was adjusted to 5 mg/mL using extraction buffer. NO generation from NOS-like activity was performed in triplicate for each sample in a 1125 μL of reaction medium containing 25 mM Tris-HCl buffer, pH 7.4, 0.2 mM CHAPS, 1 mM L-Arg, 1 mM *β*-NADPH, 1.25 mM CaCl_2_, 10 μM FAD, 10 μM FMN, 10 μM BH_4_, 1 μM/mL calmodulin, 10 μM Heme and 750 μL crude protein extract. The mixture was incubated at 37°C for 10 min. At 0 min, 375 μL of the reaction medium was taken to measure NO production by EPR employing the instrumental settings described above. At 5 min, another 375 μL of the reaction medium was used to detect NO generation by EPR. At 10 min, NO generation was detected by EPR using the left 375 μL of the reaction medium. Representative spectra from three independent measurements were shown in the results and the average EPR signal height were detected and calculated at 0, 5 and 10 min, respectively.

### cGMP detection

Extraction and detection of cGMP from *C. sinensis* pollen tubes were performed as previously described with slight modifications [30]. Briefly, 0.5 g pollen grains were incubated for 2 h, the pollen tubes were harvested, and quickly homogenized in an ice bath, after adding cooled 0.6 M PCA. The homogenate was left for 10 min and then centrifuged for 15 min at 12000 rpm at 4°C. The supernatant was adjusted to pH 4, left for 30 min in an ice bath, and then centrifuged. The supernatant was added with 50 μL Tris-acetate buffer (0.2 M, pH 7.5) and 1.7 μL of 0.5 M MgCl_2_, followed by addition of 200 μL 0.4 M ZnSO_4_ and 200 μL 0.4 M Na_2_CO_3_. The contents were vortexed for 1 min, left for 5 min at 30°C, and then centrifuged. The supernatant was prepurificated by an anion-exchange column filled with AG1-X8 400 and 0.22 μm membrane. For cGMP detection, the mobile phase was a mixture of acetonitrile and 25 mM KH_2_PO_4_ (1.25∶10, v/v) containing TBA (2 g/L) and adjusted to pH 5.5 at a flow rate of 0.6 mL/min. Samples were measured using a SHIMADZU LC-20AT HPLC system.

### Pro determination

Pro accumulation in pollen tubes was determined by the method described previously [31] and L-Pro was used as standard. Briefly, each sample (0.05 g) was incubated for 2 h at 25°C or 4°C; the pollen tubes were harvested, and extracted in 3% sulfosalicylic acid. An aliquot of each extract (2 mL) was incubated with 2 mL ninhydrin reagent (2.5% [w/v] ninhydrin, 60% [v/v] glacial acetic acid, and 40% 6 M phosphoric acid) and 2 mL glacial acetic acid at 100°C for 30 min, and the reaction was terminated in an ice bath. Toluene (5 mL) was added, followed by vortexing and incubation at 23°C for 24 h. The absorbance was measured at 520 nm.

### Statistical analysis

Each experiment was repeated at least three times. Data were analyzed using Statistical Package for Social Sciences (SPSS). The analysis of variance (ANOVA) were applied to determine the significance of the results between different treatments at the *P<0.05* level.

## Results

### Pollen germination and tube growth inhibition by cold acclimation

**Figure 1:**
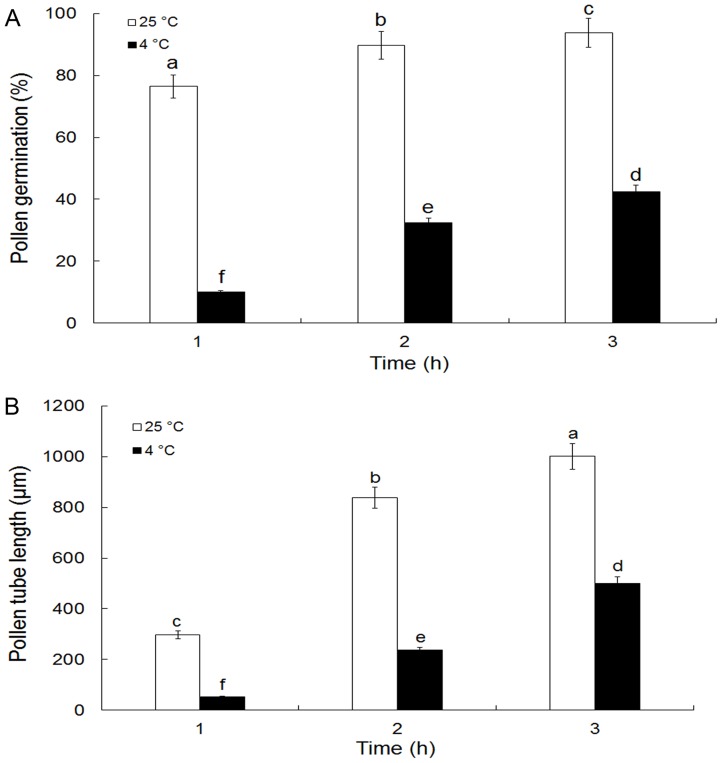
Effects of cold acclimation on *Camellia sinensis* pollen germination and tube growth. A, Typical serial course of pollen germination at 25°C and 4°C. Pollen germination was inhibited by cold acclimation. B, Typical time courses of mean pollen tube extension growth at 25°C and 4°C. Pollen tube elongation was also inhibited by cold acclimation. All data present means of three replicates ± SD. Means with different letters are significantly different at *P<0.05*.

Pollen germination and tube elongation were significantly inhibited by the cold acclimation (Fig. 1). The germination of pollen grains after incubation in control solution for 1, 2 and 3 hrs was 76.40%, 89.78% and 93.67%, respectively, whereas at the same time, only 9.97%, 32.36% and 42.51%, respectively, of pollen grains were germinated when treated with cold stress (Fig. 1A). Additionally, pollen tubes cultured under control conditions grew at an average rate of 333.40 μm h^−1^, whereas the growth rate of pollen tubes treated with cold was only 166.67 μm h^−1^. As a result, after 2 h of incubation, the average length of cold treated tubes was only 236.21 μm as compared to the 838.23 μm of control tubes (Fig. 1B).

### NO donor inhibits pollen germination and tube growth

**Figure 2:**
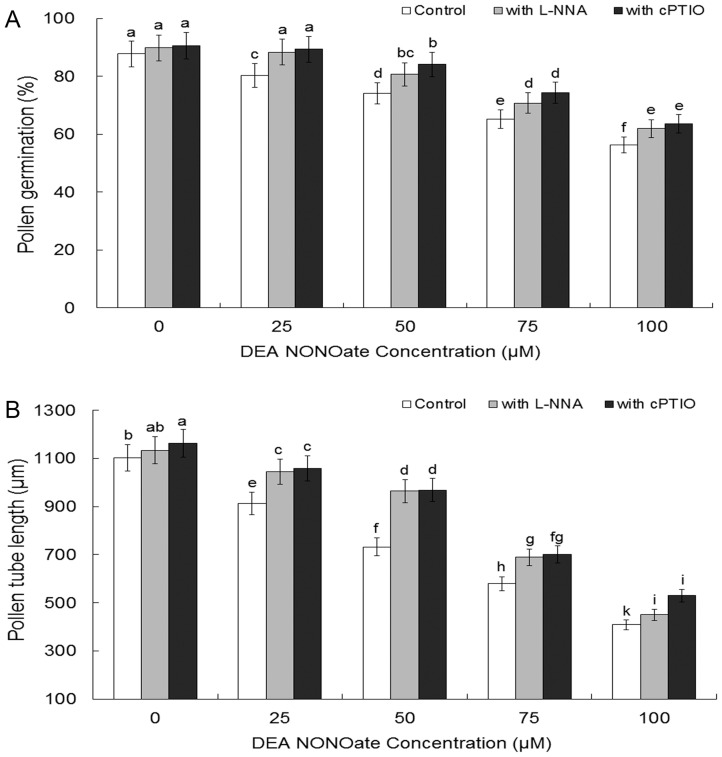
Effects of exogenous DEA NONOate on pollen germination and tube growth. Pollen germination and tube length were determined after 2 h incubation in 25°C under different concentrations of DEA NONOate, with or without 200 μM cPTIO or 300 μM L-NNA. All data present means of three replicates ± SD. Means with different letters are significantly different at *P<0.05*.

NO donor DEA NONOate significantly delayed the pollen germination and pollen tube growth in a dose-dependent manner (Fig. 2). Microscopic evaluation of pollen germination showed that 80.72%, 74.03%, 65.20% and 56.24% of pollen grains germinated when treated with 25, 50, 75, or 100 μM DEA NONOate for 2 h, respectively, whereas at this time, about 87.73% of untreated pollen grains had germinated (Fig. 2A). As shown in Figure 2B, after 2 h of incubation, the average lengths of DEA NONOate-treated tubes were 913.13 μm (25 μM), 732.5 μm (50 μM), 580.3 μm (75 μM), or 409.38 μm (100 μM), as compared to the 1102.5 μm of control tubes. Specific NO scavenger cPTIO and mammalian NOS inhibitor L-NNA were used to confirm inhibition of pollen germination and tube elongation resulting from DEA NONOate was specific to NO. Interestingly, the inhibitory effect of various levels of exogenous DEA NONOate on pollen germination (Fig. 2A) and tube elongation (Fig. 2B) were largely depressed by the simultaneous presence of 200 μM cPTIO or 300 μM L-NNA (Fig. 2). In addition, NO scavenger cPTIO or NOS inhibitor L-NNA almost had no effects on pollen germination and tube elongation in the absence of DEA NONOate.

### Inhibitory effects of Cold stress on pollen germination and tube growth are partly reversed by cPTIO and L-NNA and promoted by DEA NONOate

**Figure 3:**
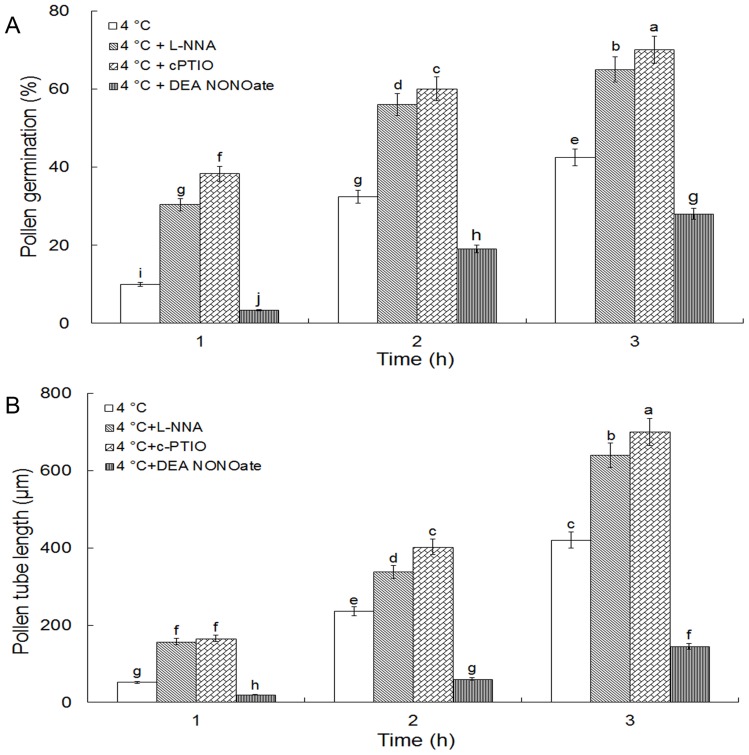
Effects of L-NNA, cPTIO, and DEA NONOate on pollen germination and tube growth under cold acclimation. Pollen germination and tube length were determined after incubating pollen grains from *Camellia sinensis* for 1–3 h in the control solution alone or containing 300 μM L-NNA, 200 μM cPTIO or 25 μM DEA NONOate, respectively in cold acclimation. All data present means of three replicates ± SD. Means with different letters are significantly different at *P<0.05*.

NO scavenger cPTIO, NOS inhibitor L-NNA and NO donor DEA NONOate were used to determine the role of NO in cold acclimation induced reduction of pollen germination and pollen tube growth. As shown in Fig. 3A, pollen germination was promoted significantly by L-NNA or cPTIO under cold stress, in contrast, delayed by DEA NONOate. The pollen germination under cold acclimation after treated with 300 μM L-NNA for 1 h, 2 h and 3 h was 30.36%, 56.34% and 60.13%, respectively; while the pollen germination under cold acclimation after treated with 200 μM cPTIO for 1 h, 2 h and 3 h was 38.31%, 64.97% and 70.16%, respectively. However, the pollen germination under cold acclimation without cPTIO or L-NNA for 1 h, 2 h and 3 h was 9.97%, 32.36%, and 42.51%, respectively (Fig. 3A). Similarly, the reduction of pollen tube elongation induced by cold stress was markedly reversed in the presence of 300 μM L-NNA or 200 μM cPTIO (Fig. 3B). In contrast, the reduction in pollen tube elongation induced by cold stress was noticeably reinforced by exogenous NO donor 25 μM DEA NONOate (Fig. 3B). Pollen tube length was 20.16 μm, 60.67 μm and 145.30 μm under 25 μM DEA NONOate at 4°C during the 1st, 2nd and 3rd hr, respectively, which was much shorter than that without DEA NONOate during the corresponding time (Fig. 3B).

### Cold acclimation induced an increase in NO generation and NOS-like activity

**Figure 4:**
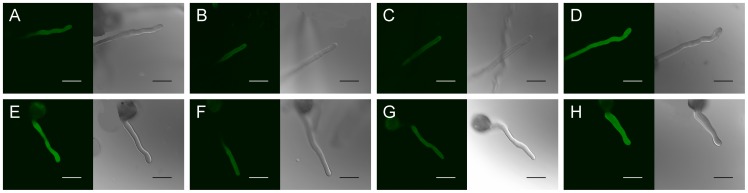
Detection of NO levels in pollen tubes using DAF-FM DA as fluorescent indicator. Bar  = 20 μm. Pollen grains of *Camellia sinensis* were incubated for 2 h in the control medium alone (A, E) containing 200 μM cPTIO (B, F), 300 μM L-NNA (C, G) or 50 μM DEA NONOate (D, H), under 25°C condition (A–D) and under cold stress (E–H), respectively. Then pollen grains grown on different treatments were loaded with 20 μM DAF-FM DA for 20 min in dark before excess fluorophore was washed out. After loading with indicator, images were acquired by CLSM Leica TCSSL. The corresponding bright field images are shown after the fluorescence images.

**Figure 5:**
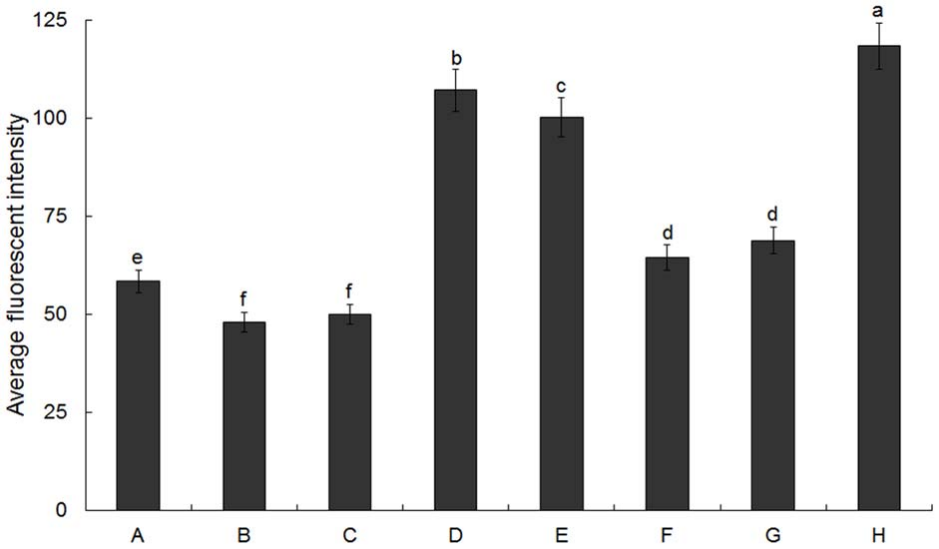
Mean relative intensities of fluorescence from NO specific indicator DAF-FM DA in germinated pollen tubes. Fluorescence images acquired from CLSM as above described (Figure 4) were analyzed and mean relative intensities of fluorescence were calculated using Image J. A–H shows the corresponding mean relative intensities of fluorescence in pollen tubes treated as above (Figure 4). Data are the means of three replicates ± SD. Means with different small letters are significantly different at *P<0.05*.

**Figure 6:**
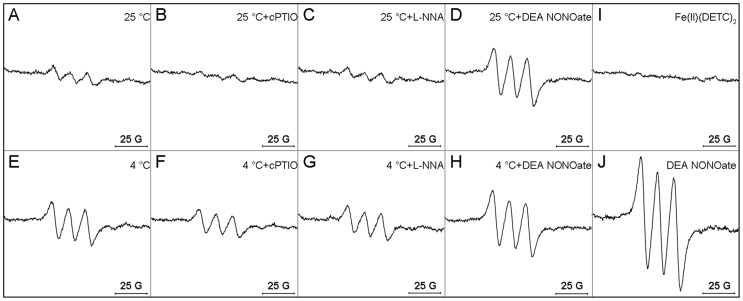
Cold-induced increase of NO in *Camellia sinensis* pollen tubes as detected by EPR. Pollen grains of *Camellia sinensis* were incubated for 2 h in the control medium alone (A, E) containing 200 μM cPTIO (B, F), 300 μM L-NNA (C, G) or 25 μM DEA NONOate (D, H), under 25°C condition (A–D) and under cold stress (E–H), respectively. Then pollen tubes grown on different treatments were harvested and NO was detected by EPR using the spin trap Fe(II)(DETC)_2_ as described in ‘Materials and Methods’. I showed typical spectra of Fe(II)(DETC)_2_ itself. J showed typical spectra of NO-Fe(II)(DETC)_2_ from 25 μM DEA NONOate. All the representative spectra are from three independent measurements.

Fluorescent probe DAF-FM DA, a membrane-permeable derivative of the NO-sensitive fluorophore 4, 5-diaminofluorescein (DAF-2) was used to analyze the presence of NO in pollen tubes. As shown in Fig. 4 and Fig. 5, the level of green fluorescence in the tubes after 2 h of culture under cold acclimation was higher than that in control (Fig. 4A, E; Fig. 5A, E). Either under control or cold condition, NO production was retarded by NO scavenger cPTIO (Fig. 4A, B, E, F; Fig. 5 A, B, E, F) or NOS inhibitor L-NNA (Fig. 4A, C, E, G; Fig. 5 A, C, E, G) in pollen tubes; in contrast, NO donor DEA NONOate induced a significant increase in the intensity of fluorescence in pollen tubes of *C. sinensis* (Fig. 4A, D, E, H; Fig. 5 A, D, E, H). Furthermore, the intensities of green fluorescence in pollen tubes under cold condition were higher than control upon exposure to NO scavenger (Fig. 4B, F; Fig. 5B, F), NOS inhibitor (Fig. 4C, G; Fig. 5C, G) or NO donor (Fig. 4D, H; Fig. 5D, H). According to recent reports, it has been revealed that DAF does not react directly with the NO free radical [32], and DAF-derived fluorescence is not specific to NO [33]. In order to further assess the role of NO during cold acclimation and confirm the changes in fluorescence were caused by NO itself, EPR analysis was also employed. The present data also showed that NO generation under cold acclimation was higher than that in control (Fig. 6A, E). Similarly, EPR signal height from pollen tubes incubating under cold stress with cPTIO (Fig. 6F), L-NNA (Fig. 6G) or DEA NONOate (Fig. 6H) was noticeably higher than that incubating under control condition (Fig. 6B–D), respectively. Fig. 6I showed the spectra of spin trap Fe(II)(DETC)_2_ and Fig. 6J showed the representative spectra of NO-Fe(II)(DETC)_2_ from DEA NONOate.

**Figure 7:**
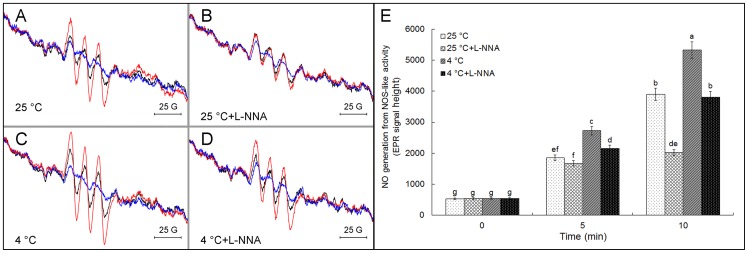
Effect of cold stress and L-NNA on activities of NOS-like enzyme reaction in pollen tubes. Pollen grains from *Camellia sinensis* were incubated under control and cold conditions for 2 h with or without 300 μM L-NNA, then the pollen tubes were collected and grounded for crude enzyme preparation according to Zhao et al. [15] with slight modification. For the detection of NOS-like activity, samples were added to a reaction mixture containing the substrate and all cofactors of the NOS reaction, and were incubated for 10 min at 37°C. NO production from NOS-like activity was detected by EPR at 0, 5 and 10 min, respectively. A, NO production from crude extracts of pollen tubes incubated at 25°C. B, NO production from crude extracts of pollen tubes treated by 300 μM L-NNA at 25°C. C, NO production from crude extracts of pollen tubes incubated at 4°C. D, NO production from crude extracts of pollen tubes treated by 300 μM L-NNA at 4°C. E showed the mean relative EPR signal height of NO from NOS-like activity, and data are the means of three replicates ± SD. Means with different letters are significantly different at *P<0.05* (E). All the representative spectra are from three independent measurements and were obtained by the accumulation of five recordings.

In addition, to further elucidate the relationship between NOS-like activity and cold tolerance on the pollen tubes of *C. sinensis*, the levels of NO generating from pollen tube NOS-like activity were measured using EPR spectroscopy. Fig. 7 showed that average NO generation from NOS-like activity of pollen tubes upon exposure to cold condition for 2 h (Fig. 7C, E) was higher than that after incubating for 2 h at 25°C (Fig. 7A, E). Interestingly, the activity of NOS-like enzyme reaction in control pollen tubes was higher than that in pollen tubes treated by mammalian NOS inhibitor L-NNA (Fig. 7A, B, E), and the increase in activity of NOS-like enzyme reaction induced by cold stress was remarkably reduced by 300 μM L-NNA (Fig. 7C, D, E).

### Reversal of cold and NO donor-inhibited pollen germination and tube growth by ODQ and reinforcement by Viagra

**Figure 8:**
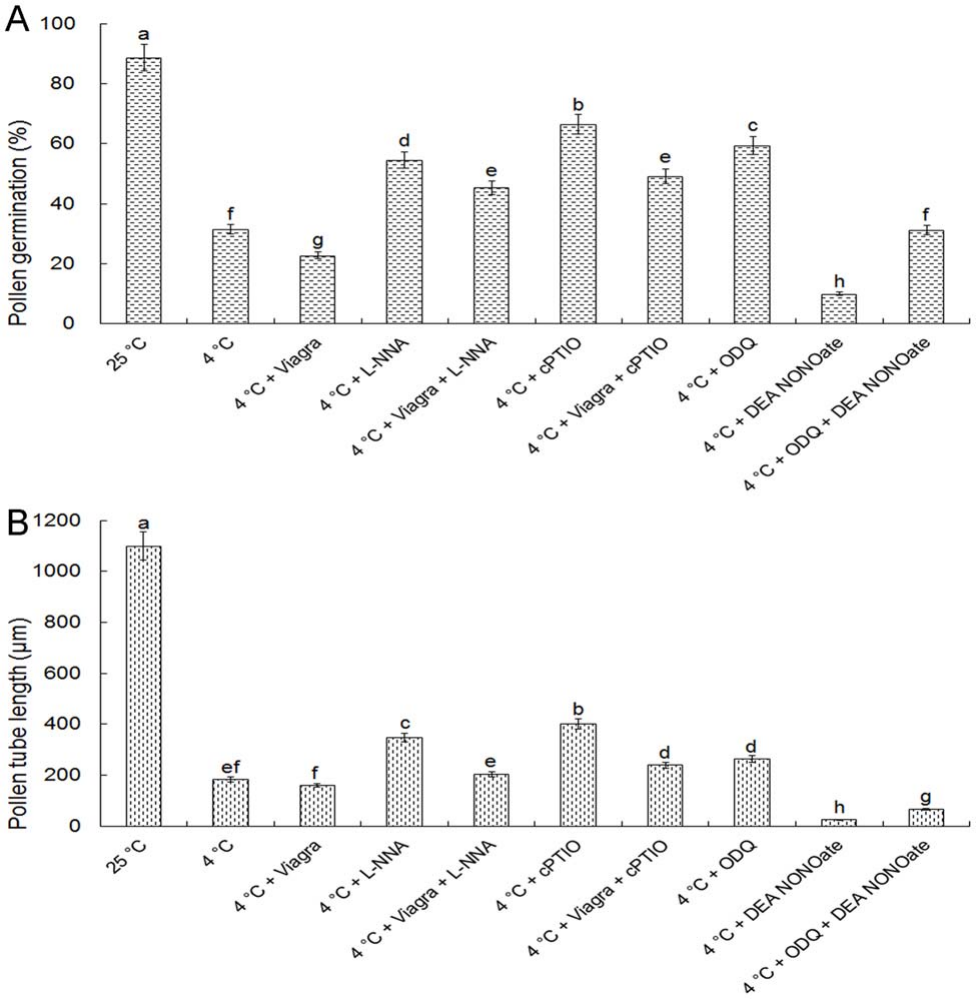
Effects of Viagra and ODQ on pollen germination and tube growth in the presence of exogenous NOS inhibitor L-NNA, NO scavenger cPTIO or NO donor DEA NONOate under cold acclimation. Pollen germination and tube length were determined after 2 h incubation. The concentrations of reagents were as follows, 80 μM Viagra, 200 μM cPTIO, 300 μM L-NNA, 50 μM ODQ and 25 μM DEA NONOate. Data are the means of three replicates ± SD. Means with different letters are significantly different at *P<0.05*.

**Figure 9:**
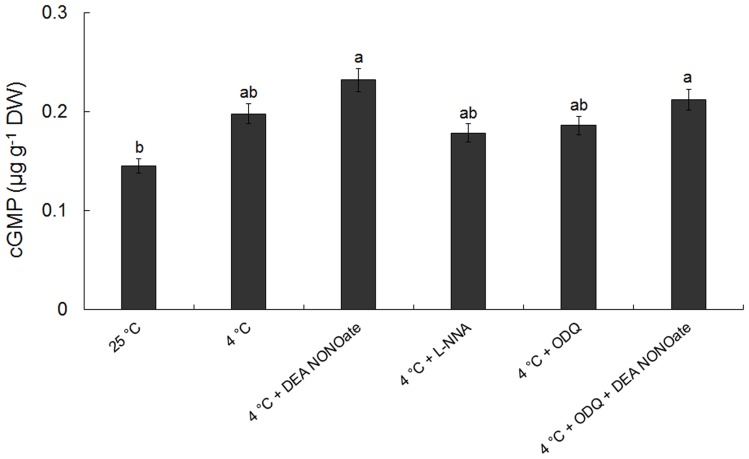
Effects of DEA NONOate and L-NNA on intracellular cGMP level in *Camellia sinensis* pollen tubes after 2 h cold treatment. The concentrations of reagents were as follows 25 μM DEA NONOate, 50 μM ODQ and 300 μM L-NNA. Data are the means of three replicates ± SD. Means with different letters are significantly different at *P<0.05*.

It was established that NO was involved in the cold inhibited pollen germination and tube growth in the above experiments. The role of cGMP in the functions of NO was further examined. The production of cGMP was blocked using ODQ, an inhibitor of GC, and the accumulation of cGMP was enhanced by PDE inhibitor Viagra. As shown in Fig. 8, pollen germination and tube elongation were lower after treatment with Viagra at cold acclimation than that treated with cold stress alone. Similarly, under cold acclimation, the pollen germination and tube elongation were lower upon exposure to Viagra with L-NNA or cPTIO than that treated with L-NNA or cPTIO alone. In contrast, pollen germination and tube growth were both higher after treatment with ODQ in cold condition than that treated only with cold stress. Meanwhile, under cold acclimation, both pollen germination and tube growth were higher upon exposure to ODQ with DEA NONOate than that treated with DEA NONOate alone (Fig. 8). In order to confirm the functions of cGMP in pollen tubes of *C. sinensis* under cold stress, cGMP content was measured using HPLC. Fig. 9 showed that cGMP content at 4°C was higher than that at 25°C, and cGMP content was much higher after treated by DEA NONOate while lower after treated by L-NNA under cold stress. Furthermore, the increase in content of cGMP resulting from low temperature or DEA NONOate was both suppressed by ODQ (Fig. 9).

### NO involvement in cold acclimation–induced Pro accumulation

**Figure 10:**
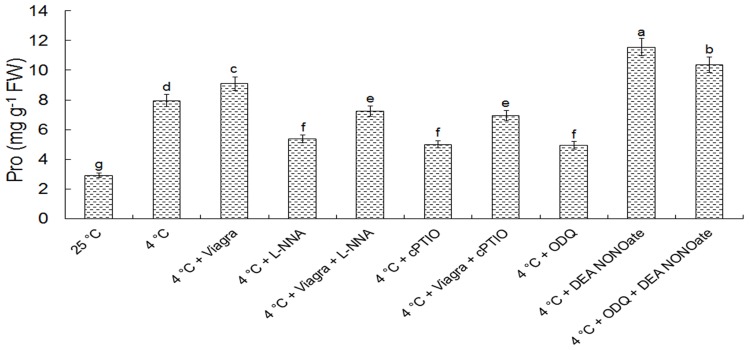
Effects of cGMP on the regulation of Pro accumulation. The concentrations of reagents were as follows 80 μM Viagra, 50 μM ODQ, 300 μM L-NNA, 200 μM cPTIO and 25 μM DEA NONOate. All data present means of three replicates ± SD. Means with different letters are significantly different at *P<0.05*.

To confirm whether the NO production from cold acclimation and its downstream cGMP signaling regulate Pro accumulation in pollen tubes of *C. sinensis*, we studied the effect of cold acclimation or exogenous NO donor in the presence of ODQ or Viagra on Pro content in pollen tubes. The results showed that Pro content of pollen tubes increased from 2.92 to 7.96 mg g^−1^ FW after 2 h of cold acclimation, compared with those grown under control conditions. Under cold acclimation, treated with 300 μM L-NNA or 200 μM cPTIO, the Pro concentration was reduced to 5.36 and 5.02 mg g^−1^ FW, respectively. However, treatment with 25 μM DEA NONOate, gave a higher Pro concentration than pollen growing in the cold environment only (Fig. 10). Higher Pro concentration was observed when 80 μM Viagra was added in the culture solution compared to exclusive treatment in the cold environment, while this finding is contrary to treatment with 50 μM ODQ in culture solution under cold stress (Fig. 10). Furthermore, the effect of 80 μM Viagra on increasing the Pro concentration in pollen tubes was partially reversed by treatment with 300 μM L-NNA or 200 μM cPTIO (Fig. 10). The effects of 50 μM ODQ on reducing the Pro concentration of pollen was also reversed by NO donor 25 μM DEA NONOate (Fig. 10).

## Discussion

Previous studies have indicated that NO plays potential regulatory roles in many developmental and physiological processes, including tip growth [24]. Meanwhile, it has been well known that pollen germination and tube growth were delayed by cold stress [34]. In our investigation, we have found that pollen germination and tube elongation in *C. sinensis* were obviously inhibited by cold temperature. Furthermore, NO donor DEA NONOate delayed the pollen germination and tube growth in a dose-dependent manner. In addition, the inhibitory effects of DEA NONOate were contracted by NO scavenger (cPTIO) and NOS inhibitor (L-NNA). These results are consistent with the results of Prado et al [21], [23] who reported NO as a negative regulator of pollen tube growth in *Lilium longiﬂorum* and *Arabidopsis thaliana*. In contrast, recent report has indicated that exogenous NO donor stimulated the *Pinus bungeana* pollen tube growth in a dose-dependent manner [24]. This study confirms that different plant species may account for differences in response of pollen tubes to NO-modulating drugs [24]. Another more interesting observation in this study was that cPTIO and L-NNA partly reversed the inhibition of pollen germination and tube growth induced by cold stress, while NO donor reinforced the cold-induced reduction in pollen germination and tube elongation in *C. sinensis*. According to these pharmacological experiments, it is reasonable to speculate that NO is not only involved in cold-induced inhibition of pollen germination and tube elongation in *C. sinensis*, but also functions as a negative regulating factor in *C. sinensis* pollen tube responding to cold stress. Our speculations are in accordance to previous report which suggested that NO has participated in another abiotic stress UV-B inhibiting pollen tube elongation in *P. tomentosa*, namely that cPTIO or L-NAME (an inhibitor of NOS) reversed the reduction of *P. tomentosa* pollen germination and tube growth induced by UV-B [26].

The involvement of NO in pollen tube growth has been reported in several recent studies in which pharmacological agents were used to alter endogenous NO levels [21], [23], [24]. In the present study, NO generation was marked weaker in the pollen tubes by adding cPTIO or L-NNA both at room temperature and cold temperature, in contrast, a sharp increase in NO was detected following the application of NO donor in pollen tubes with or without cold exposure, suggesting that exogenous NO agents effectively regulate NO level in pollen tubes of *C. sinensis*. More interestingly, as suggested in *Arabidopsis* leaves [15], we also found that NO production was elevated upon cold temperature as compared with room temperature. Combining the data, it provides strong cytological evidence that NO functions as a negative regulating factor in the polarized growth of *C. sinensis* pollen tubes responding to cold stress. Furthermore, NO level in pollen tubes has also been detected by EPR measurement confirming that cold temperature induced increase of NO production in pollen tubes.

In addition, although several candidates for NOS-like enzymes in plants remain disputable [35], mammalian NOS-like enzyme mediated NO production influencing plant development and stress responses have been reported [36], [37] and accumulating evidences have indicated that NOS-like activity involved in various processes in plants [35]. Furthermore, there have been ambiguous reports to evaluate the role of NOS-like enzyme activity in cold resistance [38], [39]. In the present study, activity of NOS-like enzyme reaction was increased noticeably under low temperature conditions compared with control in pollen tubes. Moreover, cold-induced NO production and NOS-like activities were both inhibited by specific mammalian NOS inhibitor L-NNA. Then it is plausible that NOS-like activity mediated NO production participates in the process of low temperature reducing pollen germination and tube growth in *C. sinensis*, and cold-induced NO increase is mainly produced from NOS-like activity, although findings of this study cannot directly prove NOS protein participating in *C. sinensis* pollen tube resisting to cold stress. The results were supported by previous studies which indicated that NOS-associated NO production enhanced the tolerance to cold stress both in *Chorispora bungeana* suspension culture cells [17] and pea leaves [40], while in contrast to our results, Zhao et al. [15] reported that NR-instead of NOS-associated NO increase mainly contributed to low temperature resistance in *Arabidopsis*. In our study, the effects of L-NNA or cPTIO on reversal of cold-inhibited pollen germination and tube elongation were only partial, indicating that there are other NO producing pathways and NO-independent pathways in cold signaling networks in pollen tubes of *C. sinensis*. The discrepancy in sources of NO release induced by low temperature, to some extent, may be accounted for by differences in plant species (*C. sinensis* versus *Arabidopsis*), types of tissues (pollen tube versus leaves), culture conditions and detection methods [39]. Irrespective of the discrepancy in the sources of NO production under cold stress, all the studies revealed that low temperature significantly stimulated NO production.

Recently, pharmacological and biochemical studies have defined that NO-cGMP signaling pathway participating in the regulation of cellular polarized growth [20], [21]. However, cGMP involvement in cold-induced NO production has not been deciphered so far. In our studies, Viagra as PDE-inhibitor used to stimulate cGMP level, reinforced the suppression of pollen germination and tube elongation induced by cold stress, and it also impaired the increase in pollen germination and tube growth resulting from L-NNA or cPTIO under cold stress. In contrast, GC inhibitor ODQ, at least partly, reversed the inhibition of pollen germination and tube elongation induced by cold stress with or without NO donor. These data suggested that cGMP is required, maybe partly, for cold and NO regulated pollen germination and tube growth. Furthermore, our experiments clearly showed that cold- and NO donor-induced NO stimulation resulted in cGMP increase and decrease in pollen germination and tube elongation. While NOS inhibitor induced-NO reduction resulted in cGMP decrease and increase in pollen germination and tube elongation. All above results reveal that cGMP is the downstream target of NO during resistant to cold stress in *C. sinensis* pollen tube growth, which is consistent with previous findings that NO/cGMP signaling pathway is regulating cellular polarized growth in *P. tomentosa* pollen tube [26] and *Ceratopteris richardii* spores [20].

It is well known that higher plants accumulate free Pro in response to a number of abiotic stresses such as drought, salinity, and freezing [15]. We have also found that Pro accumulation was stimulated markedly by cold stress in *C. sinensis* pollen tubes. Previous studies have revealed that Pro synthesis is associated with NO generation in *Chlamydomonas reinhardti* [13]. Our data suggested that the stimulation of Pro accumulation was enhanced by cold with NO donor such as DEA NONOate, but reduced by L-NNA and cPTIO, indicating that NO may act as a signal to evoke Pro accumulation enhancing the ability of pollen tube survive in cold stress. These results corroborate with earlier findings in Arabidopsis [15]. In addition, Viagra gave a rise to the Pro level, while ODQ resulted in prevention of the Pro concentration under cold condition. More interestingly, we also found that Viagra reversed the effects of L-NNA and cPTIO on the inhibition of Pro accumulation in tea pollen tubes, whereas ODQ restrained the Pro accumulation induced by DEA NONOate. All above results suggest that cGMP participated in the regulation of Pro accumulation in *C. sinensis* pollen tube responding to cold stress. According to previous reports, expression of *P5CS1* gene was up-regulated whereas expression of *ProDH* gene was down-regulated by cold acclimation [15]. Additionally, NO promoted the activities of P5CS1 and depressed the activities of ProDH [41], then it is plausible that cGMP might stimulate Pro accumulation via both increase of synthesis and reduction of degradation by regulating related genes expression in *C. sinensis* pollen tubes under cold condition.

**Figure 11:**
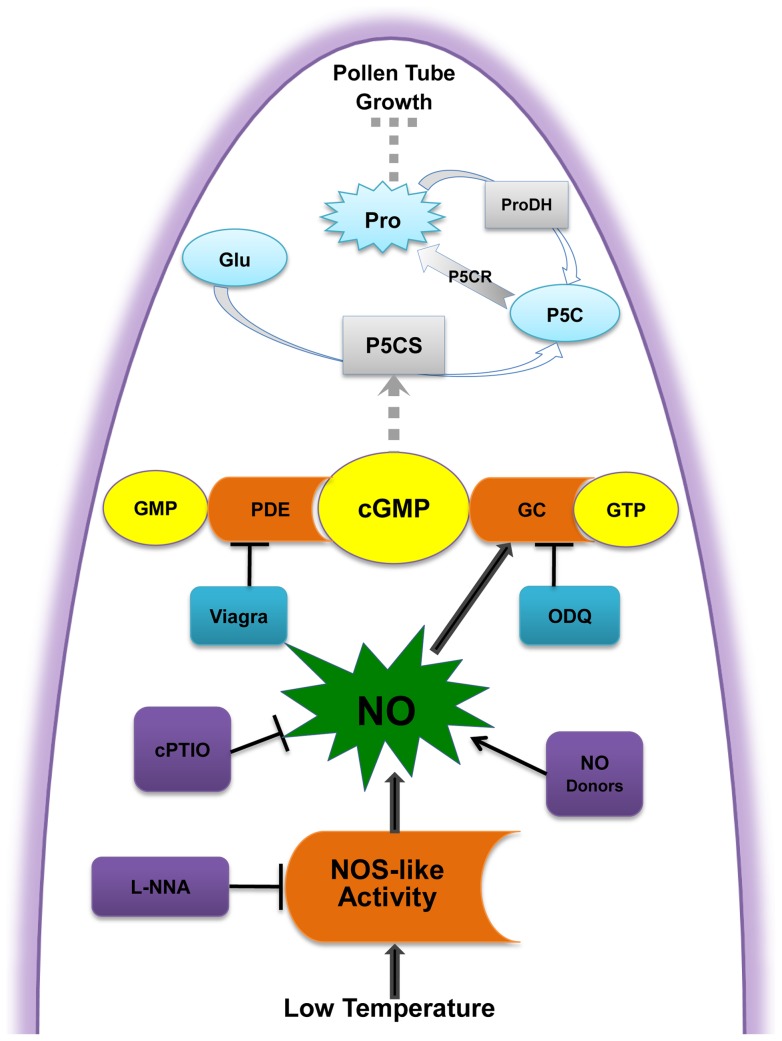
Hypothetical model showing the potential NO signaling events that participate in *Camellia sinensis* pollen tube tip growth under cold stress. This simplified model was based on the models proposed by Wang et al. [24]. Cold stress induces an increase in NO through the accumulation of NOS-like activity followed by GC stimulation resulting in cGMP enhancing. As a consequence, the proline (Pro) is stimulated. Together, these pathways lead to tip growth contraction. Black arrows indicate the links established in the induction of pollen tube development; broken arrows represent indirect or still undescribed pathways in pollen tube tip growth. NOS, nitric oxide synthase; GC, guanylyl cyclase; cGMP, cyclic guanosine monophosphate; PDE, phosphodiesterase; P5CS, delta 1-pyrroline-5-carboxylate synthase; P5CR, Pyrroline-5-carboxylate reductase; ProDH, Pro dehydrogenase; ⊥, inhibition.

In summary, it is demonstrated that a marked increase in NO generation induced by cold stress in *C. sinensis* pollen tubes was determined, at least partly, from NOS-like activity. The elevated NO may function as a signal to evoke the downstream target cGMP and hence stimulate Pro accumulation, conferring tolerance of pollen tube to cold stress in *C. sinensis* pollen tubes (Fig. 11). Overall, the results strengthen the connection between NO production and cGMP signaling pathway in the inhibitory effects of cold stress on pollen germination and tube growth, while the elaborate sources of NO in pollen tubes need further investigation.
